# Placental findings in cord accidents

**DOI:** 10.1186/1471-2393-12-S1-A5

**Published:** 2012-08-28

**Authors:** Mana M Parast

**Affiliations:** 1 University of California San Diego, CA, USA

## 

Many stillbirth cases are under- or completely un-investigated, for many reasons, including lack of consent for fetal autopsy. Placental examination is a non-invasive means, by which much information can be gained about adverse pregnancy outcomes, including stillbirth. Complete gross and histologic examination of the placenta can both point out and exclude multiple potential causes for stillbirth.

“Cord accident,” defined by obstruction of fetal blood flow through the umbilical cord, is a common ante- or perinatal occurrence. Obstruction can be either acute, as in cases of cord prolapse during delivery, or subacute-to-chronic, as in cases of grossly abnormal umbilical cords (e.g. long cord, hypertwisting, cord with true knots, entangled cord, velamentous cord, etc). In the past, this diagnosis has relied on clinical history and has otherwise been one of exclusion for the pathologist, when, following a complete autopsy and placental examination, no other cause of death is found. Since as many as 20% of normal livebirths are associated with a nuchal cord, clinical history by itself is not reliable for diagnosis of this entity. Over the past few years, we have studied the placenta in setting of cord accident, and have established histologic criteria for its diagnosis, which can identify this entity with high specificity [1,2].

Our initial study identified cord accident-related changes in large fetal vessels in the placenta [1]. These changes are all likely related to obstruction-induced vascular stasis, leading to dilated and thrombosed fetal vessels, most commonly found in the chorionic plate and large stem villous vessels [1,2]. These “minimal” criteria have a sensitivity of 62% and specificity of 79% [2]. The additional finding of fetal thrombotic vasculopathy (FTV) in sections of placental disc increases the specificity to 94%, albeit lowering the sensitivity (46%) [2]. FTV is defined by the diminuition of fetal vessels in terminal chorionic villi, leading early on to “villous stromal karyorrhexis,” with fragmented red cells and cellular debris over the receding fetal vessels, and later on, by “avascular villi,” characterized by fibrotic stroma and complete avascularity [3]. Originally suspected to be a marker for fetal and/or maternal thrombophilia, more recent studies have found it to be most commonly associated with grossly abnormal umbilical cords, which predispose to cord compression and compromise fetal blood flow [3]. Our own previous studies have shown that FTV is commonly seen in cases with gross abnormalities of the umbilical cord [4] as well as associated with fetal growth restriction, congenital heart abnormalities, and stillbirth, particularly those associated with umbilical blood flow compromise [5].

A few considerations regarding application of these criteria warrant mention here. First, these changes are characteristic of intermittent interruption of umbilical cord blood flow, and are therefore not useful in cases of acute cord accident (e.g. cord prolapse) [1]. Second, post-mortem changes in the chorionic villi can resemble changes of FTV, particularly when the placenta is retained over 48 hours following stillbirth [6]. In these settings, it is important to remember that FTV is a temporally heterogeneous lesion, while post-mortem change is uniform throughout the entire placental disc; therefore, the two are distinguishable in a careful histologic examination of the placenta [1,2,6]. Finally, due to the temporal heterogeneity of FTV, and the fact that focal FTV cannot be identified grossly, a more thorough sampling of the placental disc is warranted in the setting of stillbirth, particularly if cord accident is suspected. We routinely submit 4, instead of the usual 2, sections of the placental disc in the setting of stillbirth.
